# Using Garden Cafés to engage community stakeholders in health research

**DOI:** 10.1371/journal.pone.0200483

**Published:** 2018-08-10

**Authors:** Joyce E. Balls-Berry, Pamela S. Sinicrope, Miguel A. Valdez Soto, Monica L. Albertie, Rene Lafflam, Brittny T. Major-Elechi, Young J. Juhn, Tabetha A. Brockman, Martha J. Bock, Christi A. Patten

**Affiliations:** 1 Center for Clinical and Translational Science, Office for Community Engagement in Research, Mayo Clinic, Rochester, Minnesota, United States of America; 2 Department of Health Sciences Research, Division of Epidemiology, Mayo Clinic, Rochester, Minnesota, United States of America; 3 Department of Psychiatry and Psychology, Mayo Clinic, Rochester, Minnesota, United States of America; 4 Office of Health Disparities Research, Mayo Clinic, Jacksonville, Florida, United States of America; 5 RNeighbors, Rochester, Minnesota, United States of America; 6 Department of Health Sciences Research, Division of Biostatics and Informatics, Mayo Clinic, Rochester, Minnesota, United States of America; 7 Department of Community Pediatric and Adolescent Medicine, Children’s Center, Mayo Clinic, Rochester, Minnesota, United States of America; Telethon Kids Institute, AUSTRALIA

## Abstract

Science Cafés, informal venues to promote bidirectional dialog, inquiry and learning about science between community members, scientists, healthcare and service providers, hold promise as an innovative tool for healthcare researchers and community members to improve health outcomes, especially among populations with health disparities. However, the process of optimizing science cafés is under-studied. We describe the pilot evaluation of a series of Science Cafés, called Garden Cafés (n = 9), conducted from September 2015 through April 2016 in Olmsted County, MN and Duval County, FL to connect Mayo Clinic researchers and local service providers with the community. Selection of discussion topics was guided by a county health needs assessment, which identified community priorities. Before leaving the events, community participants completed a brief anonymous survey assessing sociodemographics and their knowledge of research benefits, readiness to participate as a partner in health research, and health and science literacy confidence. Of the 112 attendees who responded, 51% were female and 51% were Black. Respondents reported that participating in the event significantly improved (all at *p*<0.001) their understanding on all three measures. Preliminary findings suggest that Garden Cafés are an effective forum to increase community understanding and disposition to collaborate in health research, especially in members from diverse backgrounds.

## Introduction

Engaging communities in research is increasingly viewed as the cornerstone to fostering a collaborative learning healthcare system (LHS) [1] which can lead to effective translation of health research findings into clinical practice and ultimately improve the nation’s health. LHS is guided by the principle that both communities and health professionals need to be involved in healthcare; and, as an extension, in healthcare research to maximize not only the quality and relevance of the research, but also the reach and dissemination of research findings [2]. Like LHS, community-engaged research (CE) stresses the importance of engaging diverse stakeholders in the research process [2–5]. For example, while community members learn about innovations in medications for depression or treatment of heart disease, researchers learn about “real world” concerns such as the effects of these medications on a child’s performance in school. In the case of community health and research, the same concept holds true, whereby physicians, researchers, and community stakeholders learn from each other about health topics to reduce disease-related morbidity and mortality most effectively [1, 5–7].

As the complexity of clinical and translational science grows, it is critical for researchers to involve the community and patient stakeholders as active research partners [6, 7]. The National Institutes of Clinical and Translational Science fund Clinical and Translational Science Consortiums to focus on bringing research from the lab to the community to improve human health [5]. This approach is essential in enhancing the public’s trust in all phases of the research process [8].

Our academic medical center designates CE as an overarching, high-priority element for successful clinical and translational science at multiple sites. Two of our sites serve rapidly growing and diverse communities, and our third site is also growing in diversity [9]. Duval County, FL has an estimated population of 937,934 people with about 30% identifying as Black. The largest county is Maricopa County, AZ with over 4 million people and 6% being Black. Olmsted County, MN is the smallest of the three sites with a population of 144,248 people and 6% identifying as Black (i.e., African American, Somalian, and Caribbean) [8, 10–13]. In Olmsted County, MN there was a 125% increase in the number of Blacks that reside in the county since 2000. This same trend was seen with Asian (56%), Hispanic (120%), and residents of mixed race (96%) [14].

While the 1993 National Institutes of Health (NIH) Revitalization Act (amended 2017) required the inclusion of women, minorities, and subgroups into biomedical research, [11, 15] the Act did not formalize a way to educate these groups and promote such inclusion in a meaningful way. Therefore, researchers designed a new process to engage directly with the community that involved not only basic community outreach, but also incorporated reflective communication with the public to enhance dissemination, implementation, planning, and action related to health research. Using a CE approach to research (partnership between the researchers and participants) promotes equal partnerships. This was evident in studies with mental health service users and non-users by changing dynamics of their relationships from subjective to objective [16]. Strategies developed to promote bidirectional communication and stakeholder inputs into research projects were included in the Health Street model [13], “Boot Camp” Translation [16], Public Cafés [15], Science Cafés [12], and Community Engagement Studios [14]. Globally, these cost-effective approaches are useful for prioritizing community needs, clarifying research goals and offering insight from a variety of individuals [17]. Café Scientifique (Science Cafés) started as grass roots community forums to discuss research, technology and other topics of interest to the public. Cafés connected researchers with diverse stakeholders including: communities, healthcare organizations, policymakers, government organizations, and funders [18]. While the thoughtful design of these cafés can provide opportunities to engage with minorities and other underrepresented communities in the research process, the impact of this approach (i.e., co-learning) remains under-researched. There are also challenges that impede the success of Science Cafés: 1) lack of buy-in from community leaders, 2) hosting in unfamiliar locations, 3) inviting community members to engage with people they are unacquainted with, and 4) attendees don’t want to speak. In addition to these practical concerns, Science Cafés may have more impact at the individual level with more research needed on how best to use the information coming out of the café experience to influence policy changes [19]. These issues were taken into consideration in the design of the current study. Science Cafés at the University of Wisconsin Medical School are held in a public location (e.g., libraries) and allow opportunities for informal, face-to-face, bidirectional dialogues between researchers and community members on health topics [13]. Attendees at Science Cafés have reported higher levels of health and science literacy [13]. In 2015, we adapted the approach established by Ahmed et al [13] to create similar forums in Southeastern MN and Duval County, FL called Garden Cafés.

Garden Cafés serve as a viable option to educate community stakeholders on health issues and their roles in the clinical research enterprise and to offer training that will empower them to engage as equal partners in research. Alternatively, we also aim to educate researchers on best practices for respectful, meaningful engagement with the community. Connecting and engaging researchers with the community through informal dialogue about health research topics is an important avenue to building understanding and trust [13, 20] and involving the community as collaborative partners in research projects [21, 22]. The current study examines the effectiveness of the garden café model to increase community members’ health and research literacy.

## Materials and methods

This study was approved by the Mayo Clinic Institutional Review Board. We first describe the foundational work leading up to the design of the Garden Café followed by findings from a program pilot evaluation.

### Development of the garden café concept

#### Community advisory boards

Development of the Garden Café and all locally-based CE program activities are guided by our Community Engaged Research Advisory Board (CERAB) in Olmsted County, MN (formed 2012 with 18 members) and Community Research Advisory Board in Duval County, FL (formed 2010 with 15 members). Community leaders from diverse backgrounds with respect to race, ethnicity, sex, gender identity, and age [23]. Board members meet monthly and facilitate connections, provide feedback on specific projects, mentor researchers and stakeholders on the partnership process, and review pilot award proposals.

#### Community Health Needs Assessment (CHNA)

Data on local community health needs and priorities informed the Garden Café topics and all other locally based CE program activities. CHNAs were conducted in 2013 and 2016 in collaboration with several community organizations [24]. Our CE program conducted community listening sessions for diverse local stakeholder groups underrepresented in the county CHNA telephone surveys, including youth, African-American, Somali, and Latino/Hispanic, low socioeconomic status, and LGBTQ (lesbian, gay, bisexual, queer, transgender) persons [25, 26]. Sessions were developed with and conducted by community leaders trained in focus-group facilitation and analysis. Health priorities were identical in both years: mental health, chronic disease, infectious disease, and preventable disease (e.g., obesity).

#### Community outreach

Our CE program focuses on outreach, training, social media and engagement of the diverse community stakeholders described [3, 27–29]. Outreach activities occur at least twice per month at each site, most commonly in partnership with local African-American and Hispanic faith-based organizations, with attendance ranging from 6 to 2,000 individuals per event. We teach community partners to use social media to promote their organizations, to optimize health fairs, to utilize best practices for research ethics, to seek funding opportunities for community-based organizations, among others. By 2015, we obtained 144 post-event evaluations from community members aged 18–75 years; 75% were female and 71% were African-American (unpublished program evaluation). Participants rated the outreach/training as highly acceptable with respect to relevance and nearly all (96%) planned to share the information learned with other community members.

### Rationale for a science café

While our community outreach efforts have shown promising success, many of our events consisted of “one-way” communication and did not allow for meaningful stakeholder engagement and involvement in specific research projects. Researchers and communities are not familiar with appropriate and thoughtful ways to engage in a dialog about health research [3]. Thus, our next programmatic step was to adopt the Science Café as a potential forum to promote face-to-face bidirectional communication (i.e., conversations) between researchers and community members. Our objective was to provide a conduit for researchers to connect with the community to share clinical practice advances and current work that addresses stakeholder-identified needs, to offer services (e.g., blood pressure screening and fitness classes), and to discuss best practices for disseminating research results and eliminating barriers to conducting research [6, 30, 31]. The Garden Café could also provide a venue to promote educational and training opportunities and to foster new partnerships. A logic model, created to outline goals and impacts of the project, guided planning and interactions with health providers and community members. To evaluate the program, we modified the evaluation survey used in our outreach activities to include established measures of health and science literacy, knowledge of research benefits, and readiness to participate in research [11, 13, 32, 33].

### Procedures

The Garden Café was co-developed, funded and implemented with two community partners [34, 35]. With input from our community advisory boards, a welcoming community garden with 8-by-10-foot area plots were created in Olmsted County that was within 0.3 miles of the city center. The garden was accessible by mass transportation and included welcoming spaces to work, sit, and talk with fellow community members of the community (i.e., benches, tables. and chairs throughout). We also included a community bulletin board to alert people to community events including future Garden Café events. During inclement weather, we conducted similar Garden Café forums in alternative locations preferred by community partners (i.e., local coffee houses, university, and the public library). At these alternative locations, we chose relaxing and quiet spaces conducive to promoting active engagement and participation.

We promoted Garden Cafés with the help of our community partners, who invited their constituents to our events via telephone, email, flyers, social media, and face-to-face communications at centers of worship. Garden Cafés were free and open to anyone interested with healthy refreshments provided. Mayo Clinic promoted the events using resources from the Office for Community Engagement in Research (OCER). For instance, OCER used flyers, their community advisory boards and social media to increase awareness.

Garden Cafés were held from September 2015 through April 2016. Each addressed a health need identified in the CHNA [25, 26]. The planning team included staff from Mayo Clinic Community Engagement Program and Office of Health Disparities Research as well as members of the local community. Health topics addressed were: 1) fair housing, 2) mental health, and 3) preventable chronic disease. The agenda for each Garden Café included a researcher with a service provider and/or healthcare expert. The planning team selected speakers based on the CHNA priorities. Each shared 10 to 15 minutes of background on the topic (including current research findings), followed by about 45 minutes of discussion with the attendees. An OCER staff person was on hand to help facilitate the discussion. The Cafés were informal conversations between community members and researchers on a variety of health and science topics. For example, community members were able to ask researchers to explain their research programs in lay language and then provide them suggestions on study design, participant recruitment and results dissemination. At these same events, we provided the opportunity for other service-based organizations such as the public library, county health departments, and agencies focused on housing and energy assistance programs to share resources and information. Attendees were able to receive needed resources and information to help expand wellness beyond the day of the Café.

At the conclusion of the Café discussion, all participants were asked to complete an anonymous written evaluation (approximately 10 minutes). Participants did not sign a written consent form as the IRB deemed that verbal consent in the form of voluntary survey completion was adequate. We did not document the number approached to complete the survey to assess response rates, but anecdotal feedback suggested approximately 90% of attendees completed the survey.

### Measures

Survey items assessed socio-demographics including: sex, gender identity, race, ethnicity, age (18–19, 20–29, 30–39, 40–49, 50–59, 60+), and zip code (to estimate income). We conceptualized health and science literacy as confidence in ability to assess the trustworthiness of health/research information using a reliable and valid 5-item, 7-point Likert measure [13] that had been used to evaluate similar science cafés. The measure was designed to have participants rate each item twice for their level of confidence before (retrospectively) and after attending the event. In addition, we assessed respondents’ knowledge of the benefits of health research for themselves, their family, and/or community as well as their readiness to participate as a partner in a health research project [20]. Participants rated each item twice (retrospectively pre- and post-event) using a 7-point Likert-type scale with 1 = low to 7 = high levels of knowledge and readiness, respectively. Respondents were then asked if they planned to share the information learned with anyone in their family (yes/no) and in their community (yes/no). The total score was calculated by averaging non-missing items, with a possible range of 1–7. In a previous study, the total score had excellent internal consistency reliability (Cronbach’s alpha = 0.87) [13]. Cronbach’s alpha for the current sample was 0.92. Finally, the survey collected qualitative data from the participants by asking for feedback and ways to improve future events.

### Statistical and quantitative methods

Data were summarized using descriptive statistics including percentages, means, ranges, and standard deviations. Paired sample t-tests assessed change on the 5-item science and health literacy scale total score, readiness to engage in research item, and awareness of the benefits of research item. For each measure, the dependent variable was the change in post and retrospective pre-event ratings. All analyses were performed using SAS Version 9.3 (SAS Institute, Cary NC, USA). A *p*-value < 0.05 was considered statistically significant, and all statistical tests were two-sided.

Estimated household income was generated from the 2014 U.S. American Community Survey [36] by zip code (postal code) [37]. Based on the sample distribution, household income was categorized into three categories: low, middle, and high.

An open-ended item was included in the evaluation for participants to provide suggestions for improving the Garden Café experience. We used inductive thematic analysis to code participant responses [38]. Our goal was to determine additional areas of interest for future research, dissemination, and educational efforts. NVivo 10.0 (QSR International Version 10) was used to capture analysis. Initial coding was done to capture themes with any discrepancy in themes being resolved between the three coders.

## Results

### Sample characteristics

Evaluations were obtained from 112 community participants who attended at least 1 of the 9 Garden Cafés. The sample was racially and ethnically diverse; about one-third was categorized as having low income and 51% were Black. Table 1 displays selected socio-demographic characteristics of the sample.

**Table 1:** Socio-demographic characteristics of Garden Café Attendees (N = 112).

Characteristic	n (%)
Sex	
Male	50 (44.6%)
Female	57 (50.9%)
Missing	5 (4.5%)
Gender Identity	
Male	53 (47.3%)
Female	52 (46.4%)
Missing	7 (6.3%)
Race	
White	40 (35.7%)
Black	57 (50.9%)
Asian	5 (4.5%)
Native Hawaiian/Pacific Islander	3 (2.7%)
American Indian/Alaska Native	4 (3.6%)
Missing	3 (2.7%)
Ethnicity	
Hispanic	10 (8.9%)
Non-Hispanic	89 (79.5%)
Missing	13 (11.6%)
Age Range	
19 or younger	3 (2.7%)
20–29	25 (22.3%)
30–39	21 (18.8%)
40–49	18 (16.1%)
50–59	18 (16.1%)
60 +	25 (22.3%)
Missing	2 (1.8%)
Estimated Income	
Mean (SD)	$65,292 ($11,401)
Median	$65,126
Q1, Q3	$51,696,$99,145
Range	$51,809-$72,706
Income Category	
Low (< $51,809)	30 (26.8%)
Middle ($51,809–$72,706)	51 (45.5%)
High (> $72,706)	20 (17.9%)
Missing	11 (9.8%)

### Health and science literacy confidence

The mean change in pre-post ratings on each item and the total Health and Science Literacy Confidence score were statistically significant (all *p*<0.001; Table 2).

**Table 2:** Changes in ratings of health and science literacy, knowledge and readiness to participate in health research among Garden Café Attendees (N = 112).

Measure	Mean (SD) Pre-Scores^a^	Mean (SD) Post-Scores	Mean Difference (95% CI)	DF^b^
Health and Science Literacy Confidence^c^				
1. General understanding of the methods used by the scientists	4.7 (1.9)	5.7 (1.5)	1.12 (0.80, 1.45)	81
2. Ability talking about today’s topic with a health care provider	4.9 (2.0)	5.8 (1.5)	1.06 (0.69, 1.42)	84
3. Ability to tell what information is trustworthy or not on today’s topic	4.7 (1.8)	5.8 (1.4)	1.24 (0.92, 1.56)	79
4. Ability to find other sources of information on this topic	4.7 (2.0)	5.9 (1.6)	1.21 (0.89, 1.54)	83
5. Ability to speak to a scientist or health researcher	4.6 (2.1)	5.9 (1.4)	1.50 (1.12, 1.89)	85
Total Score	4.7 (1.7)	5.7 (1.3)	1.06 (0.62, 1.51)	72
Readiness Measure (single item)^d^				
Readiness to participate as a partner in a health research project	4.6 (1.9)	5.4 (1.7)	0.85 (0.50, 1.21)	87
Knowledge Measure (single item)^e^				
Awareness of the benefits of health research for self, family and/or community	5.3 (1.7)	5.9 (1.4)	0.64 (0.32, 0.95)	87

*Note*. CI = confidence interval; DF = degrees of freedom; SD = standard deviation.

^a^Ratings were based on retrospective recall of before the event.

^b^Paired sample t-tests, all *p*<0.001.

^c^Each item rated on a scale of 1 = Low Confidence to 7 = High Confidence. Total scores can range from 1–7.

^d^Rated on a scale of 1 = Low Readiness to 7 = High Readiness.

^e^Rated on a scale of 1 = Low Knowledge to 7 = High Knowledge.

Eighty-five percent of respondents reported they planned to share the information learned with family members; 63% with their community.

### Knowledge and readiness

Ratings of knowledge of the benefits of health research for self, family, and/or community improved significantly pre-post event (*p*<0.001). In addition, readiness to engage as a partner in health research also improved (*p*<0.001; Table 2).

### Qualitative results

Qualitative data were collected from participants via the evaluation surveys. Of the 112 community participants, 46 responded to the qualitative question asking for general feedback and suggestions to improve future Garden Café events. Table 3 shows selected quotes from participants. Participant responses indicated overall positive critiques of the event concept, speaker, and topic of conversation. Participants indicated a desire that the lecture be more tailored to the general public and involve more active group participation and engagement. Topics of interest for future Garden Cafés, as specified by participants, included chronic disease, access to healthcare, community- and population-specific health issues, behavioral interventions for healthy living, mental health issues, and non-medical concerns such as workplace problems and racial bias and financial obstacles for minorities.

**Table 3:** Selected feedback comments from participants.

Topic	Participant Comments
General Feedback	Very interesting; good public speaker
	Love the idea. Sign me up next season.
	Please be accountable to the community when you conduct research.
Suggestions for Improvement	Long talk, too detailed for this crowd.
	Book [on health topic] would be helpful.
	Have more of these sessions
	I would love to hear about implicit bias in the healthcare workforce.
	Better awareness of the event.
	…have more information in different language
	more of how to link others with more information
	more discussions
Suggestions for Future Topics	more information on mental health
	topics based on health insurance
	Diabetes and chronic disease.
	Alzheimer’s research & any new drugs
	Cancer
	Drug dependency and obesity
	Genealogy and health care conditions
	Business entrepreneurs
	I am interested to see as many research projects as possible for our minority communities
	Study of disease more prevalent to Somali population.
	Hypertension is important in the Somali community. We need to focus on this topic in the following meeting.
	Mental health, infectious diseases, bacterial infections
	Health-related issues regarding sedentary lifestyles
	The newer financial helps there are for the people of middle income to low middle income.

Selected feedback from participants received through an evaluation survey given after the Garden Café event

## Discussion

We found Garden Cafés to be an effective forum to increase community understanding and disposition to collaborate in health research. They were successful in eliciting participation from members underrepresented in biomedical research in Olmsted County, MN and Duval County, FL. Respondents reported that participating in the events significantly improved their understanding of health research as determined by measures of health and research literacy, willingness to participate in research and knowledge of the benefits of research. Not only were the Garden Cafés effective and well-received, they were also well attended by community members from diverse backgrounds, especially African-Americans, a group that is traditionally hard-to-reach by our academic institution. In addition, we also were able to reach a diverse group of community members with respect to gender identity, sex, income, and race/ethnicity. Our findings suggest that the Garden Cafés are feasible and effective for diverse community members.

In contrast, a study of Science Cafés held in public locations (e.g., libraries) found representation was skewed toward participants of higher SES and women [13]; race and ethnicity data were not collected. Our ability to recruit a more diverse sample might be attributed to the fact that our research team is diverse and has long-term relationships with many of the participating community groups. Our ability to easily recruit participants is also due in part to the fact that community was involved in the design of the Garden Cafés, and they provided funding, input and partnership in the development and conduct of the Cafés, including the selection of topics and presenters. Also, considerable attention was paid to making the environments of the Garden Cafés appealing to visit and easy to access (i.e., located in priority neighborhoods), with design features that were standardized and selected to be portable so they could be used in any of our locations. Collectively, these characteristics most likely contributed to our success.

To our knowledge, this was the first evaluation in the CTSA Consortium of Garden Cafés as a platform for bidirectional engagement of the community with researchers. We expanded efforts beyond traditional outreach and developed a new model for educating and engaging our community in meaningful conversations about medical research and local community health needs. Ultimately, this allowed us to create opportunities for dialogs between community members, researchers and community service providers about health and biomedical research.

While our results are promising, there are limitations to our findings. First, we used a post-test only study design with a very small set of measures that were used in similar studies (e.g., measure of health and literacy confidence). We chose this approach because this was a pilot study and we wanted to determine the acceptability and feasibility of the Garden Café to meet our goals with limited survey burden on the participants. In addition, we also did not want to ‘do research’ on our participants or change how they might behave or interact before engaging in the Café forum. Because of the post-test only design, we relied on retrospective pre-event ratings for this pilot study. However, retrospective pre-test ratings are appropriate for very short duration interventions, particularly (as in this case) where it is not feasible to administer a pre-test and the goal is to measure more general knowledge of a topic [39]. Creative ways to implement traditional pre-post designs to evaluate Garden Cafés and similar forums are needed. An alternative approach might be to provide participants with audience response keypads to provide information before and after the event or to collect additional information through qualitative observations or video of conversations, which would elicit quality information on communication without additional research burden.

A larger controlled study with a larger sample and longer follow-up are necessary to assess the potential impact of this activity on broader domains of community engagement including stakeholder collaboration and leadership on research projects. For a larger scale study, we might explore adapting our health literacy confidence measure to cover more domains of health literacy. The measures of self-efficacy, however, as the strongest predictors of behavior, are useful for the purposes of this research. We did not survey the researchers as a part of this study. A larger scale study would also benefit from evaluating researcher/community member dyad agreement on their perceptions of the quality of the conversations.

In addition, we did not assess attendees’ subsequent participation as partners in research projects. Despite this limitation, this forum led to the development of successful projects. For example, a psychiatry researcher attended and presented his research study on adolescent depression at a café meeting; and through that, met a new community contact that partnered with him on future research that was subsequently funded and is now underway [25, 26].

Other limitations of this work need to be considered when interpreting the results. One was the relatively small and possibly select nature of our sample; the geographic reach was limited to one Mayo Clinic site in Southeastern MN and Duval County, FL. Future work with larger samples, plus expansion to Maricopa County, AZ, will allow for examination of sociodemographic characteristics associated with the change in science and health literacy confidence.

Future directions will include obtaining evaluations from newly engaged sites within our academic health system. We are also exploring similar opportunities for bidirectional communication between patients and researchers; for example, leveraging disease-specific face-to-face and online Patient and Family Member Advisory Councils (PFACs), which include youth and diverse patients. Face-to-face and online discussion groups and crowdsourcing could be used to engage these patient/consumer stakeholders in bidirectional communication to identify health needs and research questions, as well as for ongoing dialog as partners in research.

As this was a pilot study, further research is needed to fully evaluate and develop a Garden Café forum that could be replicated in multiple community settings. To complement and extend the reach of face-to-face methods, our future plan is to engage virtual communities by exploring the feasibility of holding Garden Cafés via novel web 2.0 social media platforms.[29,40]. Of adults in the United States, 65% used social media sites in 2015 –primarily Facebook and Twitter [40]. From our CHNA listening sessions [25, 26], we learned underrepresented, diverse community members prefer to engage in the research process through social media. Garden Cafés offer a unique venue for further implementation and dissemination research. A concern in one location is the weather, which motivated us to explore indoor options with a similar feel. At other sites where the weather is more temperate, this may be less of a concern. Another area of opportunity is to use social media to standardize environments regardless of the location of the Café. Anecdotal feedback from community members attending the Garden Cafés and similar bidirectional forums indicated the desire for continued dialogs about research through social media. This would provide opportunities to strengthen connections and partnerships between community stakeholders and scientists. Prior work used face-to-face forums for bidirectional communications between researchers and community members, but future studies have a timely opportunity to expand these forums to innovative digital platforms. Future research should focus on the development of an evaluation process to examine the implementation and follow-up from the Garden Cafés. These domains are important for co-learning among community members as potential partners in health research. Our findings suggest the Garden Café concept is feasible and effective for communities, including those with growing diversity.

## Supporting information

S1 Text Dataset. (CSV)
